# Intracellular Recording of Human Cardiac Action Potentials on Market-Available Multielectrode Array Platforms

**DOI:** 10.3389/fbioe.2020.00066

**Published:** 2020-02-18

**Authors:** Giovanni Melle, Giulia Bruno, Nicolò Maccaferri, Giuseppina Iachetta, Nicolò Colistra, Andrea Barbaglia, Michele Dipalo, Francesco De Angelis

**Affiliations:** ^1^DIBRIS, Università degli Studi di Genova, Genova, Italy; ^2^Istituto Italiano di Tecnologia, Genova, Italy; ^3^Department of Physics and Materials Science, University of Luxembourg, Luxembourg, Luxembourg; ^4^Dipartimento di Fisica, Università degli Studi di Genova, Genova, Italy

**Keywords:** bioelectronics, meta-electrodes, intracellular recordings, multi-electrode arrays, optoacoustic poration, pharmacology

## Abstract

High quality attenuated intracellular action potentials from large cell networks can be recorded on multi-electrode arrays by means of 3D vertical nanopillars using electrical pulses. However, most of the techniques require complex 3D nanostructures that prevent the straightforward translation into marketable products and the wide adoption in the scientific community. Moreover, 3D nanostructures are often delicate objects that cannot sustain several harsh use/cleaning cycles. On the contrary, laser optoacoustic poration allows the recording of action potentials on planar nanoporous electrodes made of noble metals. However, these constraints of the electrode material and morphology may also hinder the full exploitation of this methodology. Here, we show that optoacoustic poration is also very effective for porating cells on a large family of MEA electrode configurations, including robust electrodes made of nanoporous titanium nitride or disordered fractal-like gold nanostructures. This enables the recording of high quality cardiac action potentials in combination with optoacoustic poration, providing thus attenuated intracellular recordings on various already commercial devices used by a significant part of the research and industrial communities.

## Introduction

*In vitro* electrophysiological recordings are a fundamental step in the study of neurons, cardiomyocytes, and, in general, of ion channel modulation in electrogenic cells. Although the patch-clamp concept still represents the gold standard in terms of signal quality (Hamill et al., 1981; Annecchino and Schultz, 2018), the multi-electrode array (MEA) approach has become a reference point for neuronal networks investigations (Berdondini et al., 2009) and is presently gaining significant interest from the pharmaceutical community as well (Blinova et al., 2018).

Given the broad range of different applications, MEA biosensors may present different characteristics to satisfy specific needs. For instance, the majority of neuroscientists in research labs exploit single-well passive MEAs with titanium nitride (TiN) electrodes because these represent a well-established, robust solution (Cogan, 2008; Aryan et al., 2011). For higher spatial resolution needs, high-density single-well MEAs based on silicon complementary metal-oxide semiconductor (CMOS) technology have been recently introduced (Berdondini et al., 2009; Müller et al., 2015). For commercial applications in pharmaceutics, where high parallelization and high throughput are essential, multiwell MEA plates containing several tens of MEA biosensors are used to perform parallel screening of numerous compounds.

Although they present technological differences, these commercial MEA configurations are capable of recording mainly extracellular field potentials from electrogenic cells. Thus, they are not ideal for measuring pivotal data related to transmembrane ionic currents and membrane potential. However, these data are core parameters in the study of cellular information processes, as in the case of neuronal subthreshold activity, and of the cell healthy functioning, as in the case of ionic currents modulation determined by drugs. It follows that further improvements of the MEA technology are needed for the full exploitation of this technique in applied science and commercial applications.

A significant case is represented by cardiotoxicity studies that evaluate drug candidates for potential cardiovascular liabilities, which remain today a critical factor for drug discovery and development due to the high incidence of heart-related adverse drug effects (Edwards and Aronson, 2000; Li et al., 2017; Mulder et al., 2018). In fact, the pharmaceutical industry still relies on the Automated Patch-Clamp (APC) method to assess cardiac safety, although this technique is showing low power of predictivity of cardiac drug effects due to intrinsic limitations.

In response to these needs, the scientific community is developing novel ideas to improve MEA capabilities by enabling the recording of attenuated intracellular action potentials (APs) from *in vitro* cultures, i.e., signals resembling the shape of intracellular APs but presenting lower amplitude. Attenuated intracellular APs have some limitations, as they do not provide information for example on resting potential due to MEA acquisition constraints (i.e., DC filters). However, attenuated APs can be used for the accurate measurement of ionic currents from several cells in the same culture. The most significant developments in intracellular recording on MEA have been obtained exploiting 3D nanostructures, which have the ability to tightly couple and interface with cells (Hai et al., 2010; Tian et al., 2010; Shmoel et al., 2016; Cerea et al., 2018; Dipalo et al., 2018a; Jiang et al., 2018), and using electroporation to promote cellular penetration of the 3D nanostructures (Duan et al., 2012; Robinson et al., 2012; Xie et al., 2012; Park et al., 2019). Moving toward higher spatial resolution, Abbott et al. recently demonstrated attenuated intracellular recording at network level combining 3D nanoscale intracellular electrodes and electroporation with CMOS integrated circuits (Abbott et al., 2017, 2019). However, relying on 3D nanostructures, these solutions cannot be easily translated into products to satisfy the aforementioned research and industry needs.

A very recent advancement that reached the market is represented by the LEAP (Local Extracellular Action Potentials) technology from Axion Biosystems (Hayes et al., 2019)^1^. This novel approach works on multiwell MEA plates for high-throughput applications and exploits a proprietary induction protocol to record local extracellular APs resembling intracellular signals. The technique can provide detailed information about spike deformation due to the action of compounds on the cardiac cell ion channels. However, the Axion Biosystems induction protocol cannot be repeated reliably on the same cells over several days, limiting somehow the predictivity power of the LEAP technology due to the difficulties in performing long-term monitoring of the same cells.

Recently, we have introduced the alternative concept of plasmonic optoacoustic cell poration based on laser excitation of MEA electrodes decorated with plasmonic 3D nanoantennas (Dipalo et al., 2017) or with planar metamaterials (Dipalo et al., 2018b). In particular, the use of planar porous metamaterials allowed us to obtain attenuated intracellular recordings simultaneously from thousands of cardiomyocytes cultured on already commercial high-density CMOS-MEAs (Dipalo et al., 2018b), in a completely non-invasive fashion that preserved cell integrity. Although this approach is extremely promising for high-content recordings and for specific assays that are beyond the capabilities of standard MEA or patch-clamp, the use of integrated silicon electronics may hinder the scale up to the pharmaceutical industry standards or the fast adoption from the *in vitro* electrophysiology community. Additionally, the plasmonic properties and the nano-porosity of the metamaterials pose constraints on the selection of the MEA electrodes materials.

In this work, we solve these limitations by providing evidence that optoacoustic poration can be used on the majority of electrode types used today in commercial MEA acquisition systems, from the research labs to the pharmaceutical industry. In particular, we tested successfully optoacoustic poration on (i) planar nanoporous electrodes with little or no plasmonic response by exploiting materials with low work function in water, and on (ii) non-porous electrodes with disordered fractal-like geometries of noble metals that provide high plasmonic enhancement. These findings allowed us to record high-quality cardiac attenuated intracellular APs on the two most widespread commercial MEA technologies worldwide, MEAs with titanium nitride (TiN) electrodes from Multi-Channel Systems (MCS, part of Smart Ephys, Reutlingen, DE) and multiwell MEA plates from Axion Biosystems (Atlanta, US). The former is the standard MEA device used in most research labs active in *in vitro* electrophysiology, whereas the latter is among the most common commercial MEA devices for high-throughput applications in the pharmaceutical industry. Together, these results pave the way to the quick diffusion of reliable intracellular APs recordings for cardiac biology in the academic sector and for cardiotoxicity assessment in the pharma industry.

## Materials and Methods

### hiPSC Cardiomyocytes Culture on Commercial MEAs

HiPSC-derived cardiomyocytes have been purchased from Ncardia (Cor.4U (Ax-B-HC02-1M)). Cor.4U cardiomyocytes are fully functional hiPSC-derived cardiomyocytes obtained through the *in vitro* differentiation of transgenic hiPSCs and puromycin selection technology for the resulting cardiomyocytes (Ncardia). HiPSCs were thawed and pre-cultured in a cell culture T25 flask coated with 1:100 fibronectin in Dulbecco's Phosphate Buffered Saline (DPBS) and grown overnight in Cor.4U complete medium (Ncardia) before seeding on MEA devices. This procedure allows the removal of dead cells prior to seeding and results in better assay performance. MCS MEAs and Axion 12-wells plates were sterilized with a 30 min ultraviolet exposure and subsequently treated to promote the tight adhesion of cells. The substrates were coated with Geltrex ready-to-use solution (Thermo Fischer) and then incubated for 30 min at 37°C in a humidified environment to promote the tight adhesion of the cells. Subsequently, the solution was totally removed and the cells were rapidly plated without the coating being allowed to dry. HiPSCs-derived cardiomyocytes were plated in MCS MEAs at a density of 70,000 cm^2^ and grown with Cor.4U complete medium. HiPSCs-derived cardiomyocytes were plated in Axion 12-wells plates at the density of 10,000 cells/well and grown with Cor.4U complete medium. The cardiomyocytes were grown on the devices at 37°C, 5% CO_2_, 95% air for 4 days until they reached confluence, showing spontaneous contraction activity.

### Electrophysiology Recording

All recordings from MCS-MEAs were performed at 37°C outside the incubator by a custom-made MEA acquisition system (Dipalo et al., 2017), which could record 24 out of 60 channels at 7 kHz sampling rate. This custom-made acquisition system, based on amplifiers from Intan Technologies, posed some limitations on the recording of attenuated intracellular APs. In fact, it offers a limited dynamic range from −5 mV up to 5 mV, a fixed gain of 200, and includes hardware DC filters that cannot be deactivated. The recordings on Axion 12-wells plates were recorded with the Axion BioSystems' MAESTRO system at a fixed sampling rate of 12.5 KHz. Also in this case, the employed system was an older MEASTRO version with the electronics not optimized for recording attenuated intracellular APs. The cell medium from each sample was changed ~1 h before the experiments. All data sets were analyzed by OriginLab and Matlab software.

### Laser Optoacoustic Poration

First, 5 min of unperturbed extracellular activity was recorded to characterize the culture. Then laser pulse trains were applied on the surface of both MCS MEAs and Axion plates and were used to porate the cardiomyocytes. For the laser poration, the first harmonic (λ = 1,064 nm) of a Nd:YAG (neodymium:yttrium–aluminum–garnet) solid-state laser [Plecter Duo (Coherent)] with an 8 ps pulse width and 80 MHz repetition rate was used as the radiation source for the optoacoustic poration experiments, with an average power of ~1 mW after the objective. The laser was coupled to a modified upright microscope [Eclipse FN1 (Nikon)] able to accommodate both the acquisition system from Axion Biosystems and a custom-made recording setup able to record from MCS MEAs directly on the microscope stage. A 60X immersion-mode objective (NA = 1, working distance = 2.8 mm) was used during the experiments to observe the cells on the devices and to focus the NIR laser used for poration (Figure S1). All the experiments with drugs were conducted by diluting the molecules in the culture medium and waiting 10 min before recording.

### Focused Ion Beam/SEM Cross-Section

All SEM images were acquired with a FEI Nanolab 600 dual beam system. The samples were prepared by coating the surface with 20 nm thick gold layer. Imaging was performed in immersion mode, setting the voltage at 3 kV and the current at 0.20 nA. The FIB cross sections were performed operating with a 0.23 nA Ga ion beam accelerated at 30 kV.

### Simulations

Finite Element Method implemented in the RF Module of Comsol Multiphysics was used to calculate the near-field enhancement of the porous TiN. A SEM image of a vertical cross-section (2 μm wide) of the porous TiN electrode was used as structure in a 2D simulation where the incident field was a linearly polarized plane-wave at λ = 1,064 nm. Perfect matching layer condition was put at the boundaries of the simulation region. The refractive index of TiN was taken from (Pflüger and Fink, 1997).

## Results

### Meta-Electrodes

In a previous report, we have shown the ability of nanoporous noble metal films to behave as plasmonic metamaterials able to absorb and concentrate the impinging light radiation inside the material nanogaps. These materials work as transducers by turning light into hot electrons emission and eventually into nano shockwaves that locally break the plasmatic cell membrane in a process named plasmonic optoacoustic poration (Messina et al., 2015; Dipalo et al., 2017; Zilio et al., 2017).

**Figure 1:**
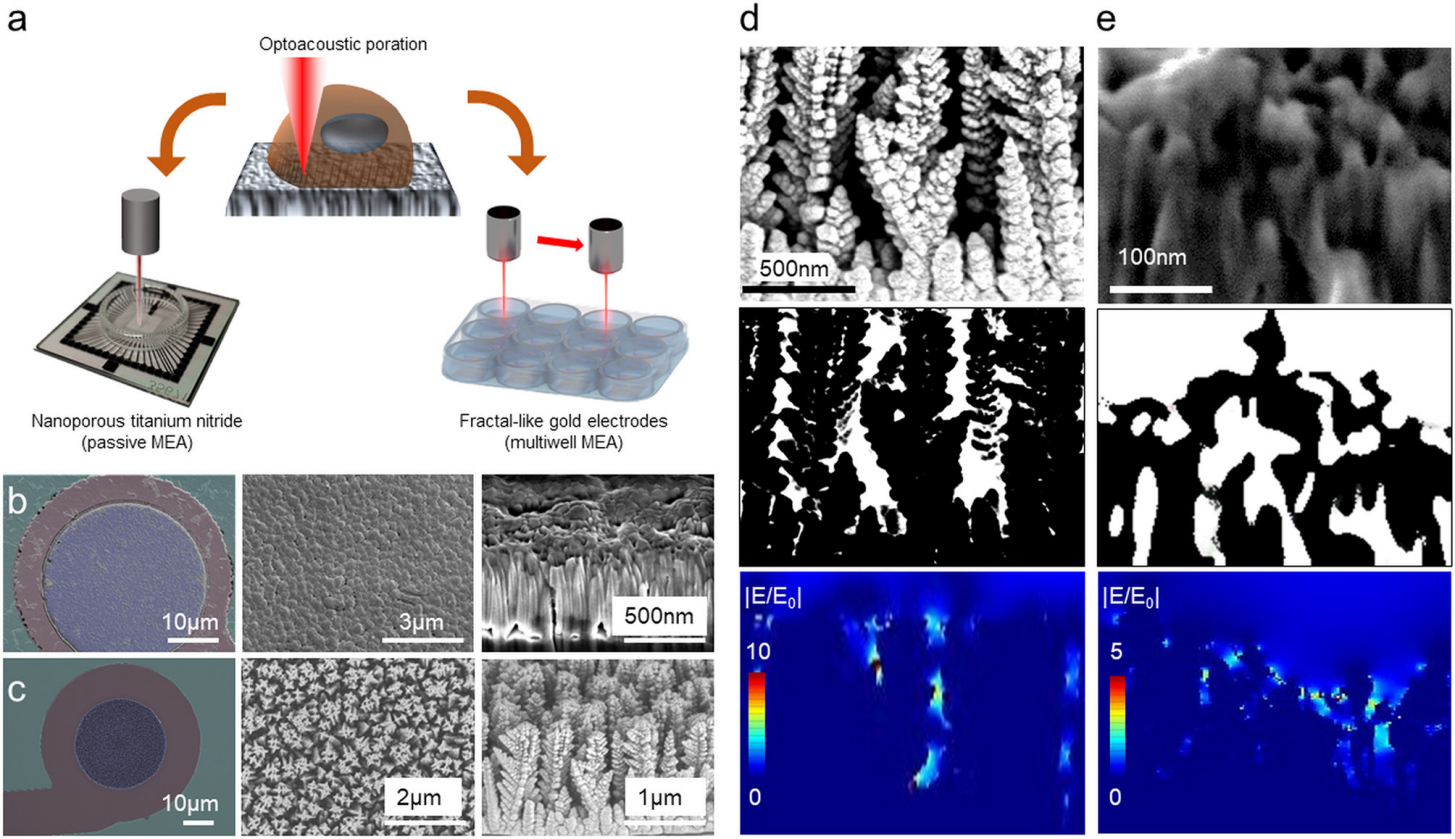
Concept of optoacoustic poration in single/multi-well configuration. **(a)** Sketch of the optoacoustic poration on nanoporous TiN electrode in single-well configuration (left) and on disordered fractal-like gold electrodes in multi-well configuration (right). **(b)** Morphological characterization of the surface of TiN electrodes from Multichannel Systems. **(c)** Morphological characterization of the surface of fractal-like gold electrodes from Axion Biosystems. **(d,e)** 2D electromagnetic simulations of the electric field distribution at the interface of electrodes from Axion Biosystems **(d)** and MCS **(e)**.

To exploit fully the potential of optoacoustic poration, here we analyze the electromagnetic response to light excitation of electrodes with material and morphological characteristics that differ from the nanoporous noble metals. Figure 1 summarizes morphological and electromagnetic characterizations of the investigated materials. In addition, Figure 1a shows schematic representations of optoacoustic poration on the two MEA configurations. The first electrode is made of planar nanoporous titanium nitride (TiN) and is used in the single-well MEAs from MCS (Figure 1a), which consist of 60 electrodes with 30 μm diameter and 100 μm pitch. Since the diffusion of MEA technology, these electrodes represented the gold standard for extracellular recordings thanks to extremely high robustness and optimal electrochemical properties (Janders et al., 1996). Figure 1b reports SEM images of the TiN electrode (on the left), a top view of the surface morphology (center) and a cross-section of the TiN layer, confirming the porosity of the material at the nanoscale.

The second electrode is made of gold and presents disordered vertical sharp nanostructures structures rather than a planar nanoporous surface. These electrodes are used in the multiwell MEA plates from Axion for high-throughput applications (Figure 1a). Figure 1c shows an image of the gold electrodes (left panel), a closer view of its surface (central panel) and a cross-sectional SEM/FIB image (right). Although they do not present porosity at the nanoscale, these electrodes offer protruding sharp tips in a fractal-like configuration that promotes engulfment by the cells and that can concentrate the electric field under illumination. Larger versions of the SEM images are available in Figure S2.

To study the optical behavior of these electrodes, we performed 2D electromagnetic simulation of the electric field distribution at the interface between electrodes surface and water (Figures 1d,e). After cross-sectional SEM imaging of both electrodes (top panels), we extracted the morphological profiles (central panels) of the materials, associating the corresponding dielectric constants of water to the empty gaps (white regions in the images) and of TiN or gold to the electrodes (black regions in the images). Then, we used these configurations for simulating the optical response under laser excitation at 1,064 nm (bottom panels). The 1,064 nm wavelength is particularly suited because it offers low phototoxicity as well as good performance with other optical characterization techniques, such as Raman spectroscopy (Yang and Akkus, 2017). As shown in Figures 1d,e, the laser radiation produces hot spots in both the materials, concentrating the electric field inside the nanogaps or on the sharp edges. For the disordered vertical gold structures (Figure 1d), the electric field presents intensities comparable to our previous work on nanoporous gold (Dipalo et al., 2018b), being thus high enough to generate local nano shockwaves for opening transient pores into cell membranes and for attenuated intracellular recordings.

For TiN electrodes (Figure 1e), given the different dielectric constants between gold and TiN, the plasmonic enhancement results to be much lower than that obtained on gold electrodes with similar porous morphology (Dipalo et al., 2018b) or with disordered vertical nanostructures (Figure 1d). However, TiN has a lower work function than gold and can generate a higher number of hot electrons under light excitation in the visible and near-infrared region (Ishii et al., 2016; Chirumamilla et al., 2017; Naldoni et al., 2017; Doiron et al., 2019; Liu et al., 2019). Since the hot electrons are responsible for generating the nano shockwave that leads to cell poration, TiN remains a promising candidate for attenuated intracellular recording by means of laser-assisted cell membrane poration.

### Attenuated Intracellular Recordings on TiN

MCS-MEAs are a reference platform in several electrophysiology laboratories in the world due to many technical advantages, like high quality extracellular recordings, versatility and high robustness. In this section, we report the results of attenuated intracellular recordings on MCS-MEAs thanks to plasmonic optoacoustic poration.

**Figure 2:**
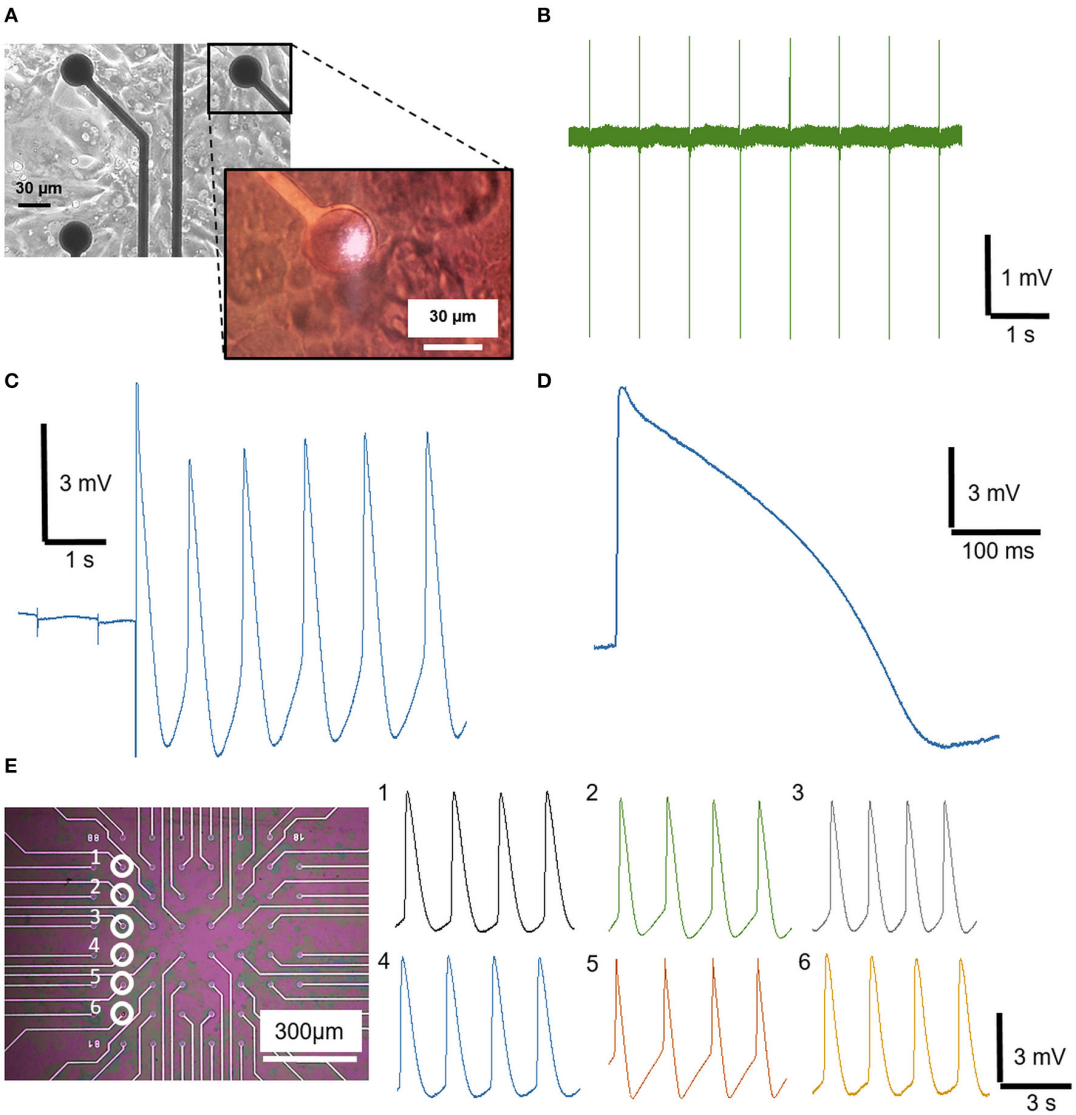
Extracellular and intracellular recording from hiPSC-derived cardiomyocytes on nanoporous TiN electrodes from Multi Channel System MEA. **(A)** Optical image of a MCS-MEA covered by a monolayer of cardiomyocytes (image taken from the bottom with an inverted microscope). The inset is an optical image acquired from above through the optics used for laser poration; it shows the laser excitation on the TiN electrode during poration. **(B)** Spontaneous extracellular field potential recording of cardiomyocytes. **(C)** Detailed view of the instantaneous transition from an extracellular to intracellular AP of cardiomyocytes after optoacoustic poration. **(D)** Magnification of an intracellular AP of cardiomyocytes. **(E)** Sequential optoacoustic poration of cardiomyocytes on six different TiN electrodes of the same MCS-MEA.

All the experiments have been conducted with human cardiomyocytes derived from induced Pluripotent Stem Cells (hiPSC-CM) purchased from Ncardia BV. We cultured the cells until they reached a monolayer conformation on the MEA devices (Figure 2A), showing a spontaneous mechanical contraction and an associated electrogenic activity (Movie S1). We then recorded the cardiac APs in the form of extracellular spikes (Figure 2B). The signals show very high signals-to-noise ratio, with an amplitude of ~3–4 mV_pp_. To achieve intracellular coupling, we then excite the TiN electrode with the ultra-fast-pulsed 1,064 nm laser (pulse train duration = 20 ms, average power = 1 mW). After laser excitation, we observe signal shapes that faithfully match intracellular cardiac APs, showing a fully positive phase followed by a repolarization step (Figures 2C,D). Thus, nanoporous TiN can effectively work as meta-electrode for plasmonic optoacoustic poration and provides high quality intracellular recordings. Despite the poor plasmonic enhancement shown in the previous section (Figure 1e), the average power required for optoacoustic poration on TiN (1 mW) is comparable with that used on nanoporous platinum or gold (Dipalo et al., 2018b). This very interesting result is to be attributed to the lower work function of TiN, which strongly facilitates hot electron emission under light excitation also at lower electric fields (Ishii et al., 2016; Naldoni et al., 2017).

In total, we applied optoacoustic poration and recorded attenuated intracellular APs on ~200 electrodes from 21 MCS-MEAs, and over 8 cell preparations. We limited the testing of optoacoustic poration to those electrodes that were covered by the cell culture and that recorded extracellular field potentials with appreciable amplitude (>100 μV), as this implied tight coupling with the cells, which is essential for successful poration. The average amplitude of the intracellular signals is ~5 mV, whereas peaks up to 9–10 mV were reached in case of optimal coupling. The measurement of amplitudes up to 10 mV was possible due the fact that, after poration and intracellular coupling, the resting potential of the cardiomyocytes pulled down the electrode potential to −5 mV, enabling a 10 mV range toward positive voltages. The time duration of the intracellular signals is ~300–400 ms, reflecting the average values of intracellular APs of hiPSC cardiac cells (Blinova et al., 2018). Having achieved high-quality recordings on MCS-MEAs with 30 μm electrodes, we also tested optoacoustic poration on MCS-MEAs with 10 μm electrodes. In this case, the improved sealing of the cardiomyocytes on the smaller electrodes could lead to intracellular signals with higher amplitudes that saturated completely the 10 mV range of the INTAN chip amplifier (Figure S3). The high seal resistance on small electrodes is specific to confluent cultures of large cells such as those of hiPSC cardiomyocytes, which cover completely the electrodes and isolate them from the cell medium (Park et al., 2019). With neurons, which are smaller than cardiomyocytes and are normally cultured at lower cell density, a lower seal resistance should be expected on planar electrodes. As mentioned in the materials and methods section, the INTAN amplifiers have limitations that prevent to exploit fully the capabilities of optoacoustic poration on MCS-MEAs. Future measurements obtained with more performing commercial MEA platforms (e.g., from MCS) could lead to higher quality recordings of attenuated APs.

The success rate of optoacoustic poration on MCS-MEAs is very close to 100%, with almost all laser pulse trains leading to intracellular recordings. To show the reproducibility of the process and thus the compatibility with network-scale recordings, we depict sequential multisite optoacoustic porations on six different electrodes of the same MEA, as shown in Figure 2E. After each poration, we could record high-quality intracellular signals from each electrode, without perturbing the activity of the whole network. In fact, the beating frequency of the porated cardiomyocytes is unaltered, as also highlighted in Movie S2, where we reproduce the spontaneous beating of cardiomyocytes before, during and after optoacoustic poration (poration event at t = 5 s). In this video, we can observe that the laser light has no effects on the porated cardiomyocyte, as expected by the intrinsic characteristics of optoacoustic poration with ultra-fast pulsed lasers (Dipalo et al., 2018b), which do not provoke thermal modulation of electrical activity like continuous-wave lasers (Yoo et al., 2019). By measuring attenuated APs on several electrodes, we could also calculate the propagation velocity in the 2D cell culture by measuring the time difference on the depolarization phases of cardiomyocytes on electrodes at specific distances. The resulting velocity is ~0.058 m·s^−1^, which is comparable to values measured for the same cells in other works (Macqueen et al., 2018). Overall, these data show that optoacoustic poration is suitable for reliable attenuated intracellular recordings on large cultures using materials with poor plasmonic enhancement, such as nanoporous TiN electrodes.

### Disordered Fractal-Like Gold Electrodes for High-Throughput Applications

Here, we provide evidence that high-quality intracellular recordings can be recorded on fractal-like gold electrodes used in one of the most popular and versatile MEA platforms for high-throughput screening already available on the market, namely the multiwell MEA plates from Axion Biosystems. We performed electrophysiological experiments with hiPSC cardiomyocytes, with the specific aim of testing the capability of optoacoustic poration in conditions closer to those applied in drug screening assays.

**Figure 3:**
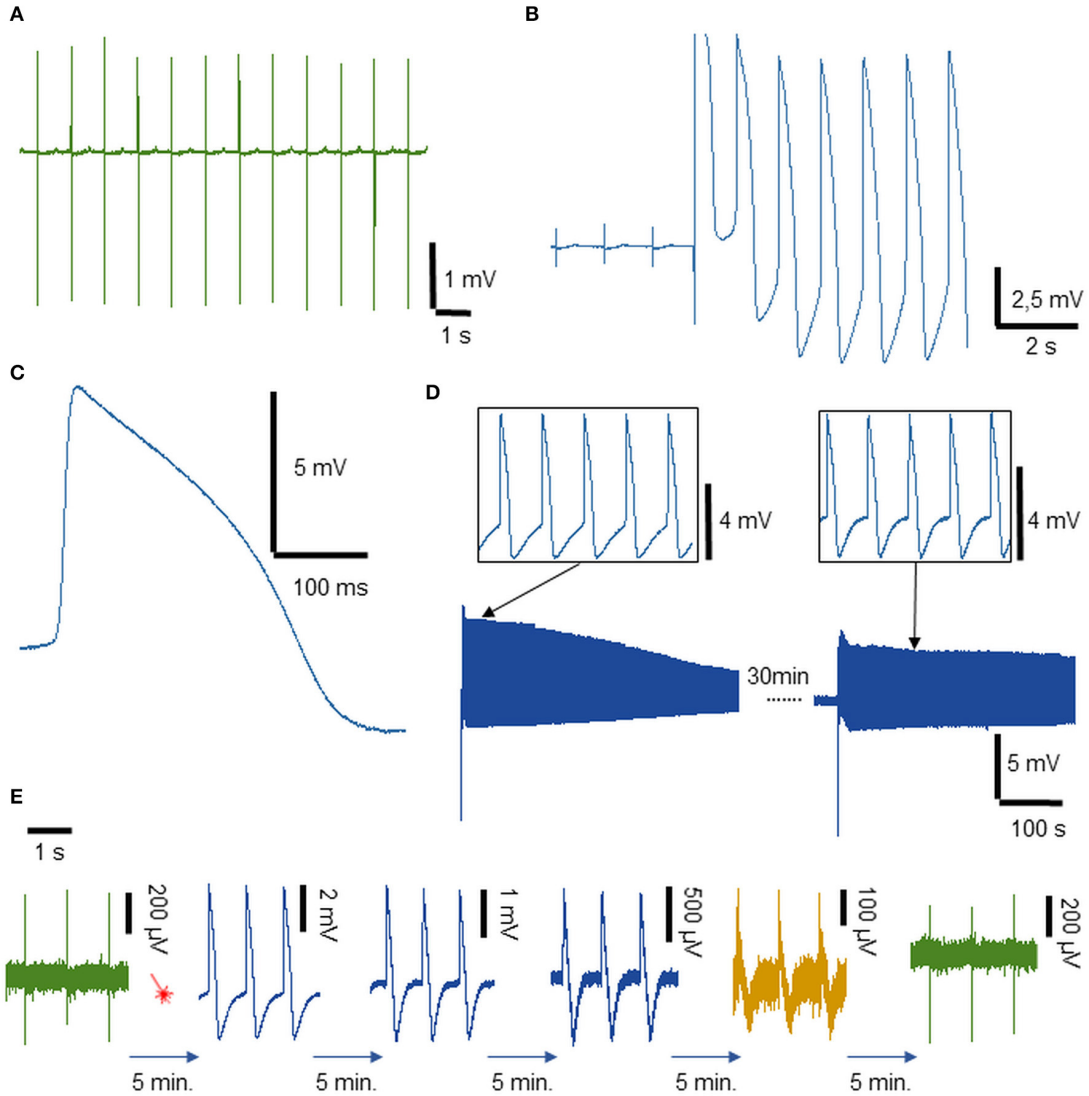
Extracellular and intracellular recordings from hiPSC-derived cardiomyocytes on disordered fractal-like gold electrodes on multi-well MEA plates. **(A)** Spontaneous extracellular field potential recordings from cardiomyocytes. **(B)** Detailed view of the instantaneous transition from an extracellular to intracellular APs of cardiomyocytes. **(C)** Magnification of intracellular AP. **(D)** Intracellular recordings of cardiomyocytes from the same electrode after repeated optoacoustic poration. Few spikes from the first trace and from the second one are reported on top of the panel. **(E)** Long-term stable recording of APs (more than 20 min): from the left, an extracellular recording followed by a laser shot (red sketch). Then, a series of intracellular recordings reported every 5 min until the cellular membrane reforms and the signal acquired again field potential characteristics.

Experiments have been conducted on these plates with the same cells and conditions used for the MCS MEA experiments. Axion Biosystems 12-well plates with fractal-like gold electrodes were used as substrates for culturing hiPSC-CMs. The Axion Biosystems' MAESTRO system has been used to acquire signals from the cardiomyocytes (Figure 3A). The MAESTRO system is capable of recording simultaneously from 768 electrodes (64 electrodes in each well for 12-well plates). We performed experiments on four 12-wells MEA plates over 2 cell preparations.

The extracellular signals show an average amplitude of 3–4 mV and a very clear T wave corresponding to the low-amplitude positive peak present after each spike. After the application of plasmonic optoacoustic poration with an average laser power of 1 mW, an instantaneous switch from extra to attenuated intracellular APs is recorded (Figure 3B). Intracellular signals show an average amplitude of 9–10 mV and a total duration of about 300 ms as depicted in the magnified view in Figure 3C. In these conditions, the success rate of optoacoustic poration was ~90%. As in the case of MCS-MEAs, we measured the propagation velocity of the cardiomyocytes also on the multiwell MEA plates from Axion Biosystems, again by measuring the time differences of depolarization phases of action potentials from distant cells (Figure S4). The velocity results to be in the range of 0.04–0.05 m·s^−1^. By translating the Axion MAESTRO acquisition system under the optical setup with a 2-axes translation stage, on each MEA plate, we were able to apply optoacoustic poration to the electrodes of six different wells (3 x 2 wells), obtaining intracellular recordings on several wells on the same multiwell MEA plate. This offers the possibility to test toxicity effects in parallel in diverse conditions (variable concentrations, different molecules…). The limitation to six wells was due to space constraints during movement under the optical microscope of the MAESTRO system, which is relatively larger than other MEA acquisition systems.

Also for multiwell MEA plates, the use of a newer acquisition platform, such as Axion Biosystems MAESTRO EDGE or PRO equipped with the BioCore V4, could lead to higher quality recordings of attenuated intracellular Aps (Axion Biosystems MAESTRO PRO)^2^.

To test the low invasiveness of our method, we performed also experiments where, after a first phase of intracellular recording, we attempted to porate again the same cell by exciting with the laser the same electrode ~30 min later. The results (Figure 3D) show that, due to the extremely controlled and localized pore opening, we are able to porate cardiomyocytes on the same electrode multiple times. Moreover, in drug development and discovery, it is also fundamental to understand the evolution of the activity of a drug on cardiomyocytes' APs in a long interval of time, extrapolating the time dose-dependence of the molecule. In Figure 3E, we show that intracellular recordings after optoacoustic poration can be stable for periods longer than 20 min. During this period, the amplitude of the attenuated intracellular APs gradually decreases because the pores in the cell membrane reseal over time (Tovar and Tung, 1992; Lin et al., 2014), with the shape of the recording gaining extracellular features again after the membrane is completely reformed. The Figure 3E reports attenuated APs from the same continuous recording, depicting APs taken every 5 min after laser optoacoustic poration. The long-term coupling and the high sensitivity allow for the detection of the dynamic temporal evolution of the signals from intracellular APs to extracellular field potentials. The time stability of the intracellular recordings is comparable with that obtained with the proprietary LEAP technology from Axion Biosystems.

### Acute Drugs Cardiotoxicity

**Figure 4:**
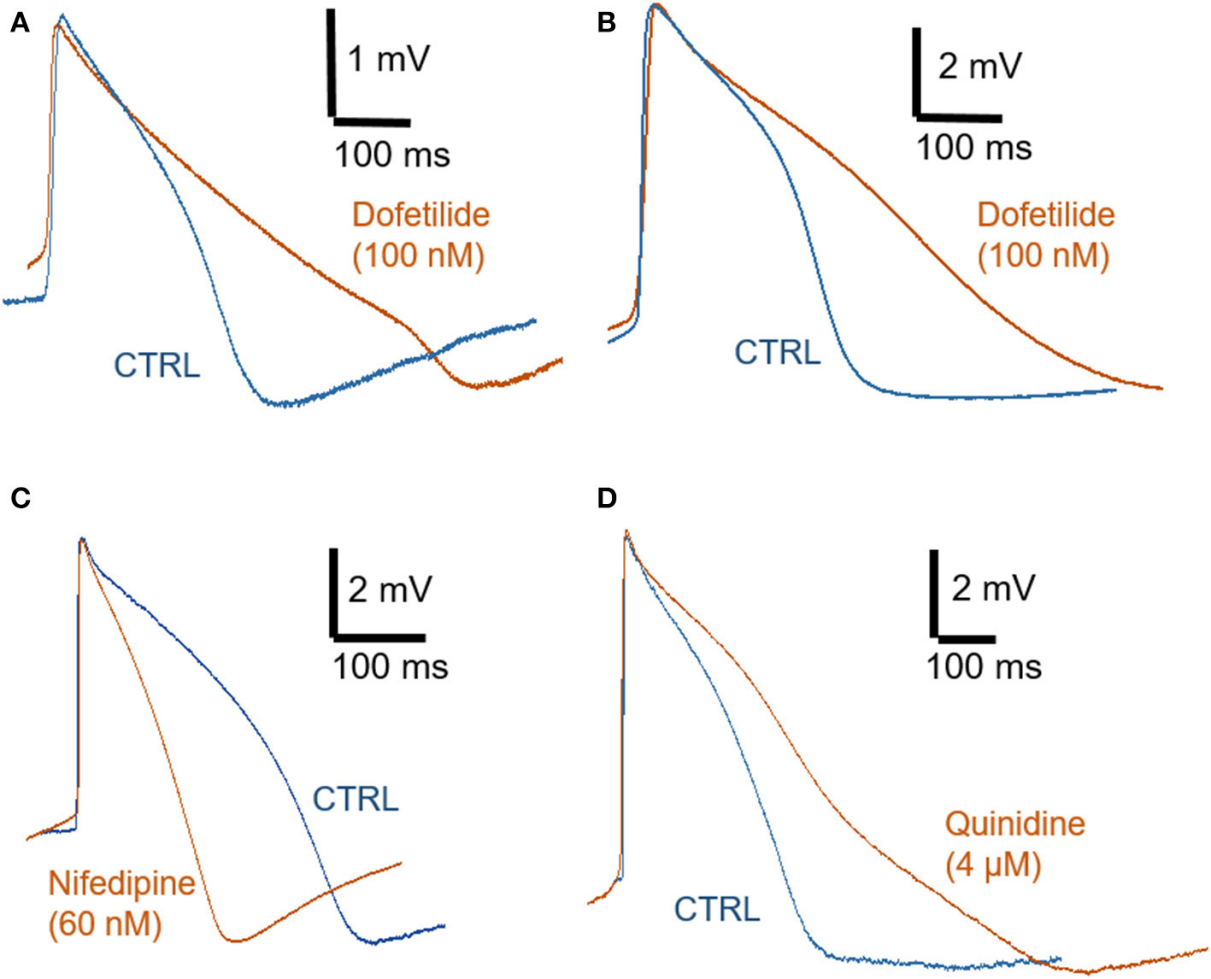
Optoacoustic poration and drug detection. **(A)** Intracellular recordings on disordered fractal-like gold electrodes on multiwell MEA from Axion Biosystems: with Dofetilide 100 nM (orange) and control (blue). **(B–D)** Intracellular recordings on nanoporous TiN on MCS-MEA: **(B)** with Dofetilide 100 nM (orange) and control (blue), **(C)** with Nifedipine 60 nM (orange) and control (blue), **(D)** with Quinidine 4 μM (orange) and control (blue).

Since the effect of many drugs in cardiac screenings is reflected in attenuated AP's shape changes, we first investigated the acute (short-term) effect of Dofetilide, a class III antiarrhythmic drug, on the shape of the cardiomyocytes' AP acquired after optoacoustic poration. Figures 4A,B illustrates attenuated intracellular APs before (blue) and after (orange) the administration of Dofetilide (100 nM) respectively on the disordered fractal-like gold electrodes of the Axion Biosystems 12-wells MEA plate and of the nanoporous TiN electrodes of the MCS-MEA. On both devices, optoacoustic poration allows highly defined intracellular signals that display the expected prolongation of the AP after the treatment with Dofetilide. In order to validate the sensibility to other drugs, we therefore analyzed the effect of other two compounds. We measured the drug-induced AP deformation after the treatment with Nifedipine (60 nM), a common calcium channel blocker that reduces the AP duration. Figure 4C shows clearly the effect of the drug on the shape of the intracellular signals before (blue curve) and after (orange curve) the treatment on nanoporous TiN electrodes. The second tested molecule, Quinidine (4 μM), induces an AP prolongation as depicted in Figure 4D, where intracellular control AP (blue curve) is overlapped with an AP post treatment (orange curve).

## Discussion

In this work, we demonstrate that cell membrane poration can be successfully achieved by laser excitation on a large family of MEA electrode configurations already offered commercially by the main MEA technology providers. In particular, we show recordings of high quality attenuated intracellular APs on MEAs with non-porous disordered gold nanostructures or with nanoporous TiN electrodes with poor plasmonic properties.

For TiN electrodes, the key factor is the low work function in water of the material, which promotes hot electron emission also at low power intensities of laser excitation. In conjunction with the porosity at the nanoscale, which enhances both cell adhesion and optical coupling, the low work function of TiN more than compensates for the poor plasmonic enhancement of the material. On the other hand, with non-porous disordered fractal-like gold electrodes, we are able to show high quality attenuated intracellular recordings on commercial multiwell MEA plates used for high-throughput applications, demonstrating that optoacoustic poration is an extremely effective technique that can have an immediate impact on pharmaceutical research. The amplitude of the recorded attenuated intracellular APs ranges from 3 to 4 mV up to ~10 mV. These values are of the same order of magnitude of those presented by several other works (Xie et al., 2012; Lin et al., 2014; Abbott et al., 2017; Hayes et al., 2019).

We obtained the intracellular signals from hiPSC-cardiomyocytes, which represent today a reference cellular model for pharmacological and toxicological studies. The intracellular recordings have been obtained by integrating ultra-fast pulsed laser excitation on unmodified commercial MEA systems used worldwide in several research laboratories, Contract Research Organizations (CROs) and pharmaceutical companies. It follows that the innovative methodology may be implemented by other bioelectronics groups by upgrading their existing MEA acquisition instruments with the proper laser source and associated optics, enabling the reliable assessment of cardiotoxicities at the industrial scale. Thus, our results pave the way to the massive implementation of the laser-based intracellular recording technique for comprehensive electrophysiological investigations of *in vitro* cell cultures. Moreover, future optimizations of optoacoustic poration may also enable intracellular recordings of neuronal cultures on commercial MEAs. Here, the main barriers to overcome would be (i) the lower seal resistance of neurons in respect to cardiomyocytes due to their smaller size and (ii) the more heterogeneous structure of neurons that could give different results if poration occurs in the soma or on processes.

Furthermore, in perspective, the enhanced electrophysiological recordings may be combined with other high performance sensing capabilities provided by the optical behavior of the electrode materials, such as Surface Enhanced Raman Spectroscopy (SERS) (Shalabaeva et al., 2017), enabling thus simultaneous multifunctional characterizations of the cells.

## Data Availability Statement

The datasets generated for this study are available on request to the corresponding author.

## Author Contributions

MD and FD conceived and planned the experiments. GM, MD, and GI carried out the experiments. NM and NC carried out the simulations and data analysis. GB and AB contributed to sample preparation and SEM imaging. All authors wrote the manuscript.

### Conflict of Interest

The authors declare that the research was conducted in the absence of any commercial or financial relationships that could be construed as a potential conflict of interest.

## Footnotes

^1^LEAP assay from Axion Biosystems. Available at: https://www.axionbiosystems.com/LEAP

^2^https://www.axionbiosystems.com/products/maestro-pro

## Supplementary Material

Movie S1 Spontaneous contraction of human-derived cardiomyocytes cultured on MEA.

Movie S2 Spontaneous beating of human-derived cardiomyocytes before, during and after optoacoustic poration (the poration occurs at *t* = 5 s).
