# Supplementary material for: Intracellular Recording of Human Cardiac Action Potentials on Market-Available Multielectrode Array Platforms
Source: Front Bioeng Biotechnol. 2020 Feb 18;8:66. doi: 10.3389/fbioe.2020.00066 (PMC7039818; doi:10.3389/fbioe.2020.00066)
Supplement: Supplementary file 1 [file Table_1.DOCX]

**S1. Experimental Setup**

**
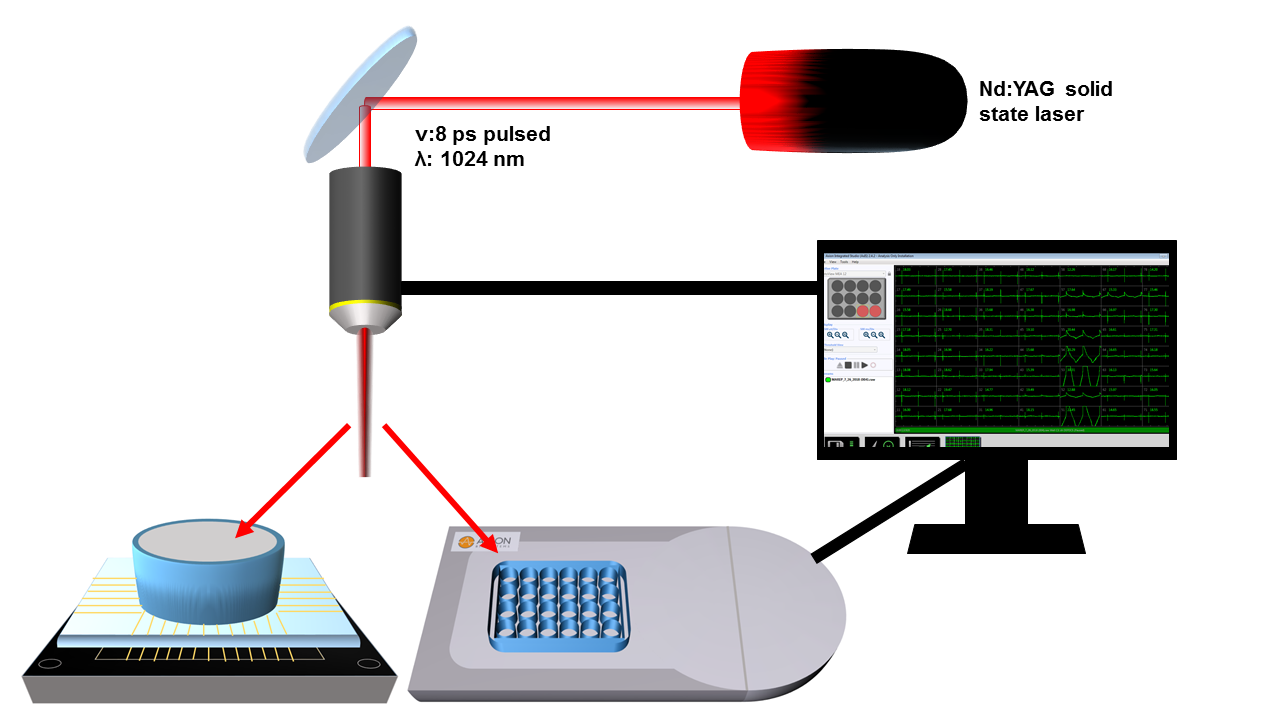
**

**Fig S1: Sketch of the setup for optoacoustic poration on nanoporous titanium nitride electrodes and on disordered fractal-like gold electrodes.**

**S2. Morphological characterization of meta-electrodes**


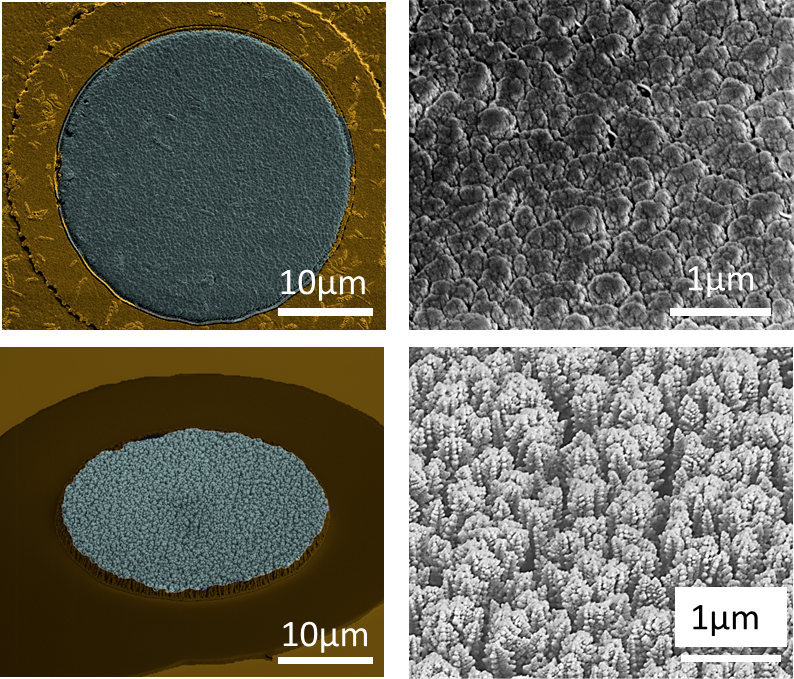


**Figure S2: SEM imaging of meta-electrodes.** The two panels on top depict nanoporous titanium nitride electrodes of MEAs from Multi Channel Systems. The two panels on the bottom depict disordered fractal-like gold electrodes of a multiwell MEA plate from Axion Biosystems.

**S3. High amplitude intracellular recordings on nanoporous titanium nitride**

**
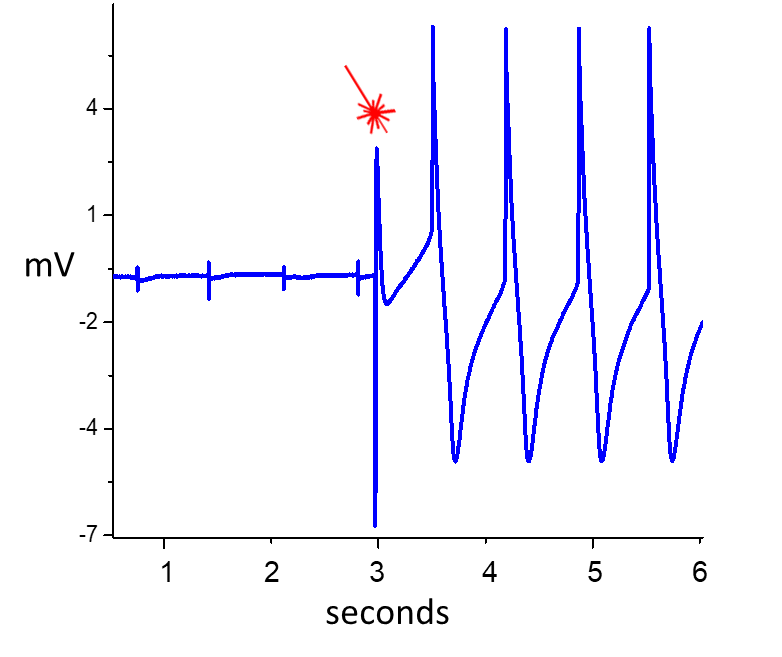
**

**Fig S3: Saturation of the MEA amplifier.** In case of electrodes of nanoporous titanium nitride with 10 um diameter, the intracellular coupling is enhanced by the tighter sealing of the cell on the smaller electrode. The intracellular signals after optoacoustic poration can saturate completely the 10 mV dynamic range of the INTAN amplifiers.

**S4. Propagation velocity measured by the action potentials**

**
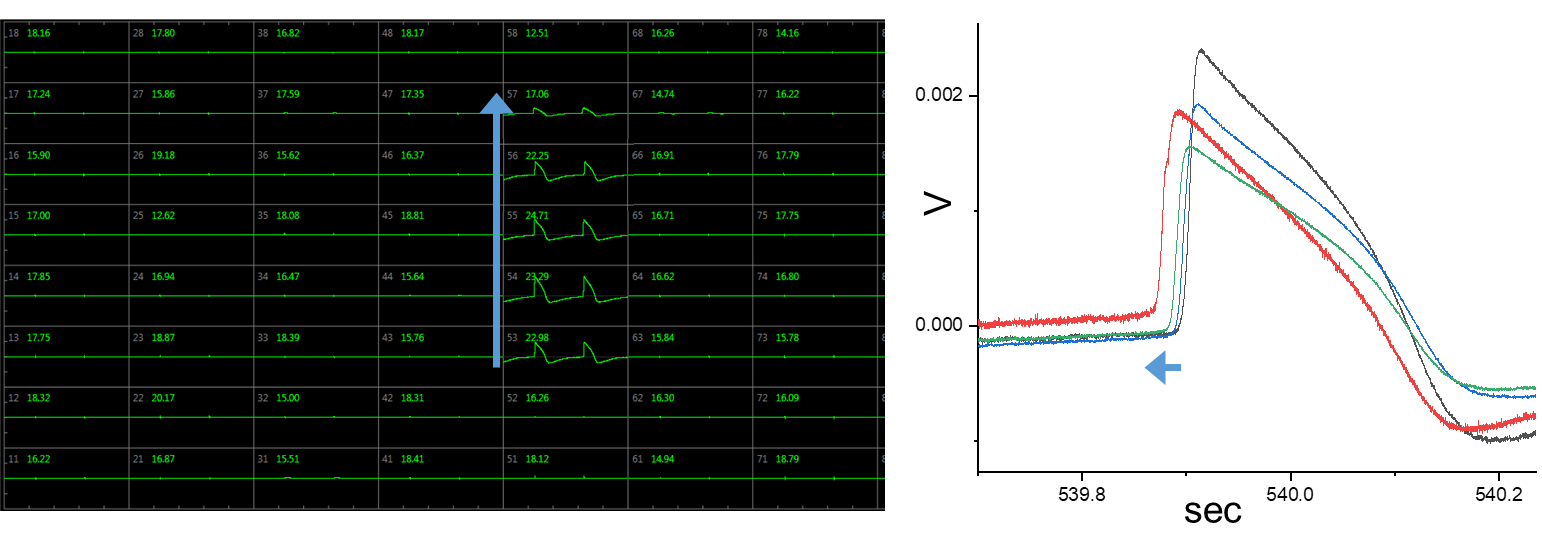
**

**Fig S4: Multiple Action Potentials recordings in the same array. Left**: screenshot of the Axion Biosystems software showing multiple action potentials recordings in the same array. The blue arrow shows the propagation direction of the spontaneous activity. **Right**: the action potentials are superimposed to calculate the time shift and thus the propagation velocity in the 2D culture.
